# Vitamin D Derivatives in Acute Myeloid Leukemia: The Matter of Selecting the Right Targets

**DOI:** 10.3390/nu14142851

**Published:** 2022-07-12

**Authors:** Ewa Marcinkowska

**Affiliations:** Department of Biotechnology, University of Wroclaw, Joliot-Curie 14a, 50-383 Wroclaw, Poland; ema@cs.uni.wroc.pl; Tel.: +48-71-375-2929

**Keywords:** acute myeloid leukemia, blast, 1,25-dihydroxyvitamin D, analogs, all-*trans*-retinoic acid, differentiation, immunomodulation

## Abstract

Acute myeloid leukemia (AML) is an aggressive and often fatal hematopoietic malignancy. A very attractive way to treat myeloid leukemia, called “differentiation therapy”, was proposed when in vitro studies showed that some compounds are capable of inducing differentiation of AML cell lines. One of the differentiation-inducing agents, all-*trans*-retinoic acid (ATRA), which can induce granulocytic differentiation in AML cell lines, has been introduced into clinics to treat patients with acute promyelocytic leukemia (APL) in which a PML-RARA fusion protein is generated by a chromosomal translocation. ATRA has greatly improved the treatment of APL. Since 1,25-dihydroxyvitamin D (1,25D) is capable of inducing monocytic differentiation of leukemic cells, the idea of treating other AMLs with vitamin D analogs was widely accepted. However, early clinical trials in which cancer patients were treated either with 1,25D or with analogs did not lead to conclusive results. Recent results have shown that AML types with certain mutations, such as isocitrate dehydrogenase (IDH) mutations, may be the right targets for differentiation therapy using 1,25D, due to upregulation of vitamin D receptor (VDR) pathway.

## 1. Introduction

Acute myeloid leukemia (AML) is a malignancy of the myeloid blood lineage, characterized by the rapid growth of abnormal cells (blasts) in the bone marrow. The blast cells overgrow bone marrow, preventing normal blood cell production, and expanding to circulation, where they are unable to function properly. Since leukocytes produced in bone marrow belong to the immune system, every AML is accompanied by an immune deficiency resulting in vulnerability to infections. In addition, inability to produce appropriate amounts of red blood cells and platelets results in anemia and bleeding [1].

The primary goal in the treatment of AML is an elimination of leukemic blasts. However, chemotherapy blocks not only the proliferation of blasts, but also the proliferation of immune cells, an essential step in immune cells’ activation. Therefore, chemotherapy-induced immunodeficiency adds to leukemia-induced immunodeficiency [2].

AML is a relatively rare disease which constitutes about 1% of all malignancies. It is a disease common in elderly people and very rare in children, with about 25% of cases diagnosed among adults aged 65–74 years and 34% among these aged 75 and older [3]. AML is the most heterogeneous hematologic malignancy with about 200 known underlying mutations [4]. For more than 40 years, all AML patients have been treated using standard intensive chemotherapy, combining anthracycline and cytarabine. For patients who responded with complete remission after intensive chemotherapy, stem cell transplantation was their treatment of choice [3]. However, it should be remembered that most AML patients are elderly and not fit for either intensive chemotherapy or stem cell transplantation. Understanding disease heterogeneity has allowed for the development of lower-intensity and more targeted treatments for elderly patients who are unfit for intensive treatments [3].

**Figure 1:**
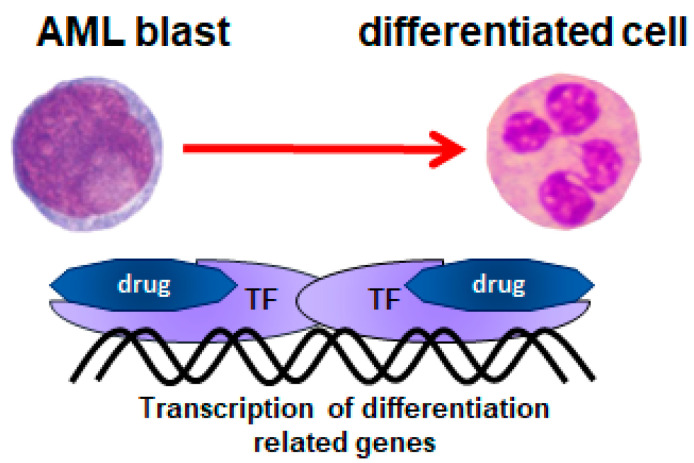
The general idea of differentiation therapy. AML—acute myeloid leukemia; TF—transcription factor.

Leukemic blasts are inhibited in their differentiation by either genetic abnormalities or by gene-expression anomalies. These cells do not express the proteins important for the function of their normal counterparts. Therefore, finding a method of forced differentiation of leukemic blasts seemed to be a particularly attractive solution for AML patients. Differentiation therapy is based on forced transcription of the genes that are crucial for the function of normal counterparts to leukemic blasts. This concept has been based on the findings concerning normal hematopoiesis, where the eventual cell fate is governed by spatiotemporal fluctuations in transcription factor concentrations, which either cooperate or compete in driving target-gene expression [5]. Some of these transcription factors have critical roles in lineage selection [6], while others govern cell cycle exit and expression of lineage-specific genes [7]. There are several reasons why transcription factors in leukemic blasts do not operate properly: one of them may be epigenetic silencing of the gene, while the others are mutations [8,9]. The general idea of this type of therapy is presented in Figure 1.

## 2. All-*trans*-Retinoic Acid (ATRA)

Acute promyelocytic leukemia (APL) is a subtype of AML characterized by uncontrolled expansion of blasts, which are blocked at the promyelocytic stage of hematopoiesis. Cytogenetically, APL is characterized by a translocation between the long arms of chromosomes 15 and 17 [t(15;17)]. This aberration leads to the fusion between the promyelocytic leukemia gene (*PML*) located on chromosome 15q21, and the retinoic acid receptor α gene (*RARA*) from chromosome 17q21, forming the chimeric oncogene *PML-RARA* [10]. In its first description in 1957, APL was considered to be the most malignant form of AML, accompanied by severe bleeding and very short survival time [11]. Retinoic acid receptor α (RARα) is a nuclear receptor activated by two metabolites of retinoic acid (RA): all-*trans*-RA (ATRA) or 9-*cis*-RA. When dimerized with a retinoid X receptor α (RXRα), it binds to response elements located in the promoters of target genes, activating their transcription. In the absence of the ligand, RARα/RXRα induces chromatin condensation and repression of transcription [12]. Activated RARα/RXRα regulates many genes crucial for myeloid differentiation, for example these encoding transcription factors PU.1 and CCAAT/enhancer-binding proteins α and ε (C/EBPα and C/EBPε) [13,14,15].

Fusion protein in APL contains the *N*-terminal part of PML protein and the C-terminal part of RARα, and in terms of function it influences transcription. ATRA at physiological concentrations is unable to release complexes of co-repressors from PML-RARα, leading to transcription blockade [16]. It has been noticed, however, that supra-physiological concentrations of ATRA are able to cause the exchange of co-repressors to co-activators, activating the transcription of genes responsible for granulocytic differentiation [17]. Importantly, the blasts lose their immortality following differentiation processes, and start to die by apoptosis [18]. In fact, surprisingly, the very first demonstration that ATRA is capable of inducing granulocytic differentiation was in using HL60 cell line, which is not an APL subtype [19]. However, in clinical situations only patients who have the t(15;17) mutation respond to ATRA treatment, which was reported for the first time in 1988 [20]. Despite experiencing rapid remission when treated with ATRA alone, the patients suffered from relapse within 6 months. Arsenic trioxide (ATO) used in the patients who relapsed after initial treatment with ATRA had significantly improved results [21,22]. The mechanisms of beneficial action of ATO in APL are SUMOylation, ubiquitination, and eventual degradation of the PML part of the fusion protein [23]. Most of the current protocols combine ATRA, ATO, and cytostatics, such as cytarabine or idarubicin. Using these protocols, complete remission (CR) can be achieved in 90–100% of patients, while overall survival (OS) rates can be achieved in 86–97% of patients [24]. This highlights the great success of differentiation therapy, indicating that the proper combinations of drugs with complementing mechanisms of action are needed.

There were many attempts to widen the success of ATRA therapy beyond APL subtypes of AML. There were some clinical trials in which ATRA was added to chemotherapy [25]. Analysis of one trial suggested that the beneficial effects of ATRA were restricted to the subgroup of patients with a mutated nucleophosmin 1 (NPM1) gene, and without fms-like tyrosine kinase 3 (FLT3)-internal tandem duplication (ITD) [26]. Unfortunately, in other trials this beneficial effect was not observed [27,28]. In fact, in some cases ATRA may even worsen the patient’s situation, as it was in the case of the patient with t(4;15)(q31;q22) translocation, resulting in the expression of the TMEM154-RASGRF1 fusion protein. This patient was treated with ATRA and died from rapid disease progression, which was related to ATRA-induced activation of RARγ, a RAR isoform responsible for hematopoietic stem cell renewal and proliferation [29].

## 3. 1,25-Dihydroxyvitamin D_3_ (1,25D)

The possibility to use 1,25-dihydroxyvitamin D_3_ (1,25D) in differentiation therapy originated from a study published in 1981, where mouse myeloid leukemia cells exposed in culture to 1,25D were induced to differentiate into functional macrophages [30]. This discovery was extended to human HL60 cells soon after [31,32]. The beneficial actions of 1,25D against AML were also presented in mouse models of this disease [33,34].

The idea to use 1,25D against cancers originated from epidemiological studies. These studies indicated an association between an increased risk of developing colorectal cancer and a low level of 25D in the blood [35,36], as well as an increased risk of developing breast cancer and a low blood level of 25D [37,38]. The role of 1,25D in solid cancers has been discussed in a detailed manner in another paper from this Special Issue [39].

**Figure 2:**
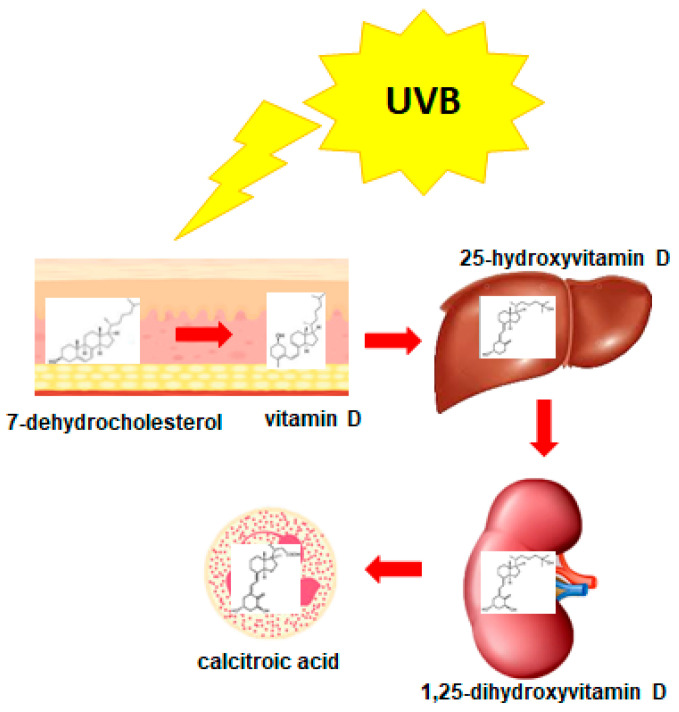
Vitamin D metabolism. Vitamin D is produced in human skin from 7-dehydrocholesterol following exposure to UVB. Then, vitamin D undergoes two hydroxylations: at C-25 in the liver by 25-hydroxylase, and at C-1 in the kidneys by 1α-hydroxylase. Degradation of 1,25-dihydroxyvitamin D (1,25D) into inactive metabolite (calcitroic acid) occurs by hydroxylation at C-24 by 24-hydroxylase in all cells which express vitamin D receptor (VDR).

1,25D is an active metabolite of vitamin D, which, despite being named a “vitamin”, is a steroid hormone [40]. It is produced by the human body from cholesterol and, similarly to other steroid hormones, its effective concentration is strictly regulated by feedback mechanisms. Vitamin D is produced from 7-dehydrocholesterol in human skin when exposed to UV light. Activation of vitamin D is controlled by cytochrome P450 mixed-function oxidases (CYPs) and occurs in two steps: 25-hydroxylation followed by 1α-hydroxylation [41]. The first stage of activation occurs in the liver, where vitamin D undergoes enzymatic hydroxylation by 25-hydroxylase (CYP2R1/CYP27A1), converting it to 25-hydroxyvitamin D (25D). Then, 25D is transported to the kidneys, where it undergoes further hydroxylation at C-1 by 1α-hydroxylase (CY27B1) and results in the formation of the active metabolite, 1,25D. Hydroxylation of 1,25D at carbon atom C-24, catalyzed by 24-hydroxylase of 1,25D (CYP24A1), is the first step of its inactivation. Since the gene encoding CYP24A1 is the most strongly upregulated 1,25D target, it provides negative feedback to the activity of 1,25D and controls the effective concentration of this highly active compound [42]. The metabolism of vitamin D is presented in Figure 2.

**Figure 3:**
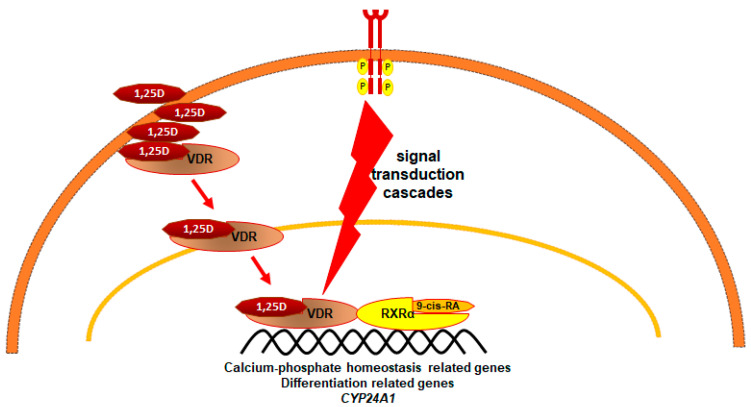
Vitamin D receptor (VDR) pathway. 1,25D translocates through the plasma membrane and binds to its receptor in the cytosol. Ligated VDR is transported to the cell nucleus, where it dimerizes with RXRα. VDR/RXRα complex binds to response elements in the DNA to regulate transcription of target genes. Signal transduction from membrane receptors participates in the activity and stability of VDR [47,48,49].

The major and most well known role of 1,25D is to maintain the calcium phosphate homeostasis of the organism [43], but it is well-documented that 1,25D regulates other vital processes, such as differentiation and proliferation of the cells [40]. The vitamin D receptor (VDR), similarly to RARα, is the nuclear receptor which after binding its ligand translocates to the cell nucleus, where it acts as a ligand-activated transcription factor. VDR, after binding 1,25D, heterodimerizes with RXRα in order to regulate transcription of target genes [44]. There are hundreds of VDR-regulated genes [45], many of them responsible for maintaining calcium phosphate homeostasis [43]; however, there are also many genes involved in immune functions, exemplified by CD14, encoding a macrophage co-receptor for bacterial LPS [46]. The overview of 1,25D/VDR intracellular pathway is presented in Figure 3.

Encouraging results of in vitro and murine studies prompted some clinical trials conducted with small groups of patients with myelodysplastic syndrome (MDS) and AML [50,51]. In these trials either 1,25D or its precursor 25D were used, but results were variable and inconclusive. In general, combination treatments resulted in better outcomes than 1,25D alone [52,53]. For example, the combination of 1,25D, AraC, and hydroxyurea resulted in complete or partial responses in 79% of patients with AML [54].

## 4. Low-Calcemic Analogs of 1,25D

One of the problems with therapeutic uses of 1,25D is its calcemic action and possible consequences of hypercalcemia [55]. In fact, in some of the very few clinical trials in which 1,25D was used against MDS, patients suffered from hypercalcemia [56,57]. The symptoms of hypercalcemia might vary from mild to severe, such as nausea, fatigue, loss of appetite, arrhythmia, kidney failure, calcification of soft tissues, and decalcification of bones [58]. This problem may be overcome by use of low-calcemic analogs which are available from many laboratories [59].

Many analogs of 1,25D have been synthesized with intention to split its activities. The idea was to reduce calcemic actions and retain pro-differentiating activities. Despite the fact that numerous analogs have been available for over 30 years, it is still not clear how the split of these activities is obtained [60]. The most puzzling is the fact that there is only one VDR which mediates calcemic and pro-differentiating actions. It is possible, then, that different analogs activate different intracellular signaling pathways, but it is still not clear how this would be achieved [61].

Analogs of 1,25D have been modified in one or more sites of the structure of the parental compound [59]. Some modifications are minor, but some change the structure substantially [62]. It is noteworthy that not only analogs of 1,25D can be used as agonists of VDR: lithocholic acid (LCA) is a natural ligand, and a very weak agonist of VDR. Modifications of LCA structure can substantially increase the pro-differentiation potency of LCA, without affecting calcium phosphate homeostasis [63,64,65]. Unfortunately, the clinical trials using analogs of 1,25D were also far from these for ATRA in APL [66].

## 5. The Heterogeneity of AML

The most likely source of failure in differentiation therapy using 1,25D and analogs lies in the heterogeneity of AML. There are two systems of AML classifications, the French–American–British (FAB) system from 1976 [67], and the World Health Organization (WHO) system from 2008 [68]. In the FAB system, all AMLs are divided into 8 groups, based predominantly on the cell morphology and cytochemical staining [69]. The later WHO system divided AMLs into 7 groups. This system is much more complicated because it is based on a combination of clinical characteristics, morphology, immunophenotype, cytogenetics, and molecular genetics of the blasts. It takes prognostic factors known to affect the treatment and the outcome of the leukemia into consideration [68,70]. Neither of these classifications is ideal; therefore, there are some attempts to make amendments [3]. APL is an M3 subtype according to FAB, and belongs to group 1 according to WHO (AML with recurrent genetic abnormalities). In addition to variability of driver mutations in AML, there is also intrinsic heterogeneity in each patient resulting from clonal diversification of blasts [71]. The most frequent mutations in AML have been identified and are used to guide treatment and predict outcome. These are *NPM1* mutations, DNA methyltansferase 3A (*DNMT3A*) mutations, *FLT3* mutations, isocitrate dehydrogenase (*IDH*) mutations, ten-eleven translocation 2 (*TET2*) mutations, runt-related transcription factor (*RUNX1*) mutations, CCAAT enhancer binding protein α (*CEBPA*) mutations, additional sex comb-like 1 (*ASXL1*) mutations, mixed lineage leukemia (*MLL*) mutations, protein p53 (*TP53*) mutations, c-Kit mutations, or *PML-RARA* translocation t(15,17)(q22;q12). Out of these examples, only the M3 subtype, characterized by *PML-RARA*, is susceptible to ATRA-based differentiation therapy.

## 6. AMLs Resistant to 1,25D

**Figure 4:**
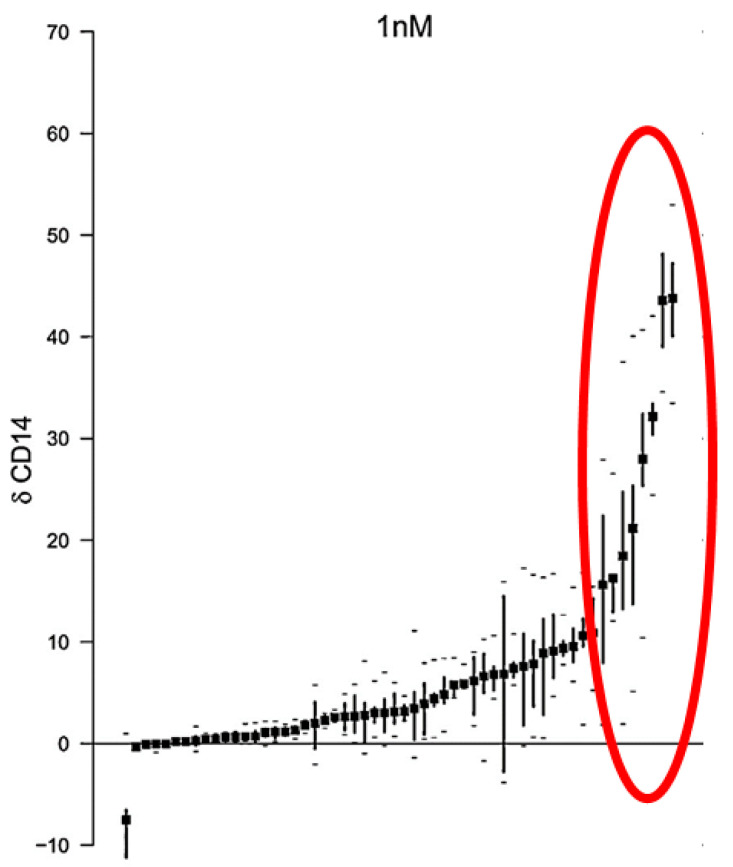
The monocytic differentiation of blasts from AML patients in response to 1 nM 1,25D and 1 nM analogs. The blasts of AML patients were isolated from peripheral blood and exposed to either 1 nM 1,25D or to one of the eight low-calcemic analogs at 1 nM concentration. Mean gain in expression of CD14 cell surface antigen for each patient is presented as a dot (•). Quartiles of response are marked by vertical lines, while minimum and maximum values are marked by dashes (–). Red oval surrounds the data from patients whose blasts were susceptible to 1,25D and to analogs. Adapted from [72].

The lessons learnt from ATRA therapies prompted studies focused on identification of AML subtypes sensitive and resistant to 1,25D-induced differentiation. In one study, the majority of patient’s blasts did not respond to 1,25D or to the analogs with monocytic differentiation [72]. Figure 4 shows that only about 25% of the blasts were responsive. The correlation study performed using blasts isolated from AML patients indicated that blasts carrying FLT3 mutations are resistant to 1,25D and to its analogs [73]. Surprisingly, available cell lines which carry FLT3 mutations, MV-11 and MOLM-13, are responsive in vitro to 1,25D and to analogs [74]. There are some possible explanations for this phenomenon, including that the correlation observed was not due to a causal implication, or that the cell lines grown in vitro for many years had changed their phenotype due to epigenetic changes.

The data from ALM patients indicate that *VDR* expression levels positively correlate with patients’ survival. VDR controls the stemness of blast cells and promotes their differentiation [75].

The cell line which was found to be completely resistant to 1,25D-induced cell differentiation is KG1 [76]. This cell line has very low expression of *VDR* gene as compared to other AML cell lines, very low levels of VDR protein, and almost no response of VDR target *CYP24A1* [74]. KG1 cells originated from 8p11 myeloproliferative syndrome, a blood disease which rapidly develops into AML [77]. KG1 cells are characterized by a chromosomal translocation where FGFR1 oncogene partner 2 (*FOP2*)—the fibroblast growth factor receptor 1 (*FGFR1*) fusion gene—encodes a constitutively active fusion protein FOP2–FGFR1. This fusion protein constitutively activates signal transducer and activator of transcription (STAT) 1 and STAT5 [78,79]. Disruption of this fusion gene restored expression of VDR gene, and sensitivity to 1,25D-induced monocytic differentiation [80]. Whether or not a similar situation exists in patients with 8p11 myeloproliferative syndrome remains to be elucidated. The obstacle to study this is that the mutations observed in this syndrome are not routinely tested in patients with AML [81,82].

## 7. AMLs Sensitive to 1,25D

It seems obvious that in order to benefit from immuno-stimulating activity of 1,25D in patients with AML, it is necessary to define the subtypes of the disease which are sensitive to 1,25D-induced differentiation.

An interesting observation was made about AML cases with IDH mutations. These mutations result in the production of the (R)-2-hydroxyglutarate (2-HG), which causes a hypermethylation, and dysregulates hematopoietic differentiation. One specific mutation in IDH is a R132H substitution. AML blasts with this specific mutation have been shown to have certain transcription factor genes upregulated when compared to the cells without this mutation. *CEBPA* gene and resulting protein C/EBPα were enriched in mutated cells. Interestingly, AML blasts harboring this particular mutation were more responsive to ATRA than blasts with wild-type (wt) IDH. Moreover, a cell-permeable form of 2-HG sensitized wt-IDH1 AML cells to ATRA-induced myeloid differentiation [83]. AML cells with IDH-R132H mutation also have higher levels of VDR and RXRα proteins than the cells with wt-IDH. Consequently, these cells respond better to 1,25D than wt-IDH cells, and even better to the combination of 1,25D and ATRA [84].

In fact, combination therapy using 1,25D and ATRA was postulated long ago, when VDR protein was found to be upregulated in ATRA-treated Kasumi-1 cells [85]. However, the regulation of *VDR* gene by ATRA is quite complex, and depends on the cell context [74]. This is because an abundant and unligated RARα acts as a suppressor of *VDR* transcription, while following ligation with ATRA or with RARα agonists, starts to act as an activator [86]. This shows that patient-tailored combination therapy should be advised.

**Figure 5:**
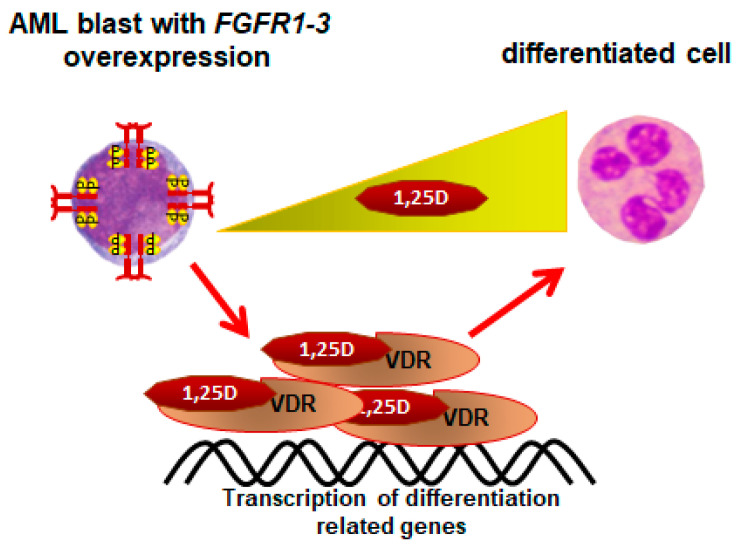
Differentiation of AML blasts with *FGFR 1-3* overexpression. The AML blasts with overexpression of *FGFR 1-3* produce more VDR protein than wild-type cells, and therefore are more susceptible to 1,25D-induced differentiation.

Another recent observation about the sensitivity of AML cells to 1,25D concerns the cells with overexpression of *FGFRs*. In addition to chromosomal translocations, *FGFR* genes may be affected by other mutations. Gene amplification of *FGFR1* was discovered in squamous cell lung cancers and estrogen-receptor-positive breast cancers, while *FGFR2* in some gastric cancers and in some triple-negative breast cancers [87,88]. There are data that indicate that the *FGFR1* gene is amplified in some cases of AML also [25]. In AML cell lines, overexpression of *FGFR1-3* caused enhanced sensitivity to 1,25D-induced differentiation, due to enhanced expression of *VDR* gene (Figure 5) [89]. Whether a similar regulation exists in the AML blasts of patients remains to be studied.

The *FGFR* family contains five genes, out of which four encode transmembrane tyrosine kinase receptors that exist in multiple splicing variants. Binding of the ligand to FGFRs results in a dimerization of these receptors and transphosphorylation of their tyrosine kinase domains [90]. As a result, FGFRs activate different signaling cascades including mitogen-activated protein kinase (MAPK), phosphatidylinositol 3-kinase (PI3K), and phospholipase Cγ (PLCγ) [91]. It has been shown in the past that activating some of the MAPK pathways, namely JNK and Erk-1,2 pathways, enhances 1,25D-induced cell differentiation [92,93]. In contrast, constitutively active FGFRs, such as in FOP2–FGFR1 fusion kinase, cause downstream activation of signal transducer and activator of transcription (STAT) pathways [94]. Our unpublished data indicate that activation of STAT1 is responsible for low *VDR* expression.

## 8. Conclusions

AML is a disease of the elderly, and the proportion of older people is increasing steadily in modern societies. The current estimate of the probability of developing cancer is one in two for people born after 1960 [95], and despite the fact that AML is a relatively rare malignancy, its numbers will grow in the near future. For more than 40 years, all AML patients have been treated using standard intensive chemotherapy, but intensive chemotherapy cannot be used for elderly people. When chemotherapy is given to elderly patients, they are often unable to tolerate it. Consequently, there is a need for gentler drugs for use alone or in a combined treatment. Differentiation therapy provides a much milder approach to treating malignancy, and should be advanced. However, the great success of ATRA-based differentiation therapy against APL has shown that this type of therapy must be targeted to molecular lesions susceptible to differentiation-inducing drugs. Recent data indicate that similarly to ATRA, 1,25D, or its analogs should be applied only to these patients who are likely to respond. Recent advances in next-generation sequencing, transcriptome analysis, immunophenotyping, and multiparameter flow cytometry will provide the means to delivering patient-tailored and tolerable differentiation therapies in the near future.

## References

[1] Pelcovits A., Niroula R. (2013). Acute Myeloid Leukemia: A Review. Rhode Isl. Med. J..

[2] Weycker D., Barron R., Kartashov A., Legg J., Lyman G. (2014). Incidence, treatment, and consequences of chemotherapy-induced febrile neutropenia in the inpatient and outpatient settings. J. Oncol. Pharm. Pract..

[3] Newell L.F., Cook R.J. (2021). Advances in acute myeloid leukemia. BMJ.

[4] Padmakumar D., Chandraprabha V.R., Gopinath P., Vimala Devi A.R.T., Anitha G.R.J., Sreelatha M.M., Padmakumar A., Sreedharan H. (2021). A concise review on the molecular genetics of acute myeloid leukemia. Leuk. Res..

[5] Friedman A. (2007). Transcriptional control of granulocyte and monocyte development. Oncogene.

[6] Brown G., Hughes P., Michell R., Rolink A., Ceredig R. (2007). The sequential determination model of hematopoiesis. Trends Immunol..

[7] Theilgaard-Mönch K., Pundhir S., Reckzeh K., Su J., Tapia M., Furtwängler B., Jendholm J., Jakobsen J.S., Hasemann M.S., Knudsen K.J. (2022). Transcription factor-driven coordination of cell cycle exit and lineage-specification in vivo during granulocytic differentiation: In memoriam Professor Niels Borregaard. Nat. Commun..

[8] Heidari N., Abroun S., Bertacchini J., Vosoughi T., Rahim F., Saki N. (2017). Significance of Inactivated Genes in Leukemia: Pathogenesis and Prognosis. Cell J..

[9] Nie Y., Su L., Li W., Gao S. (2021). Novel insights of acute myeloid leukemia with CEBPA deregulation: Heterogeneity dissection and re-stratification. Crit. Rev. Oncol. Hematol..

[10] Rowley J., Golomb H., Dougherty C. (1977). 15/17 translocation, a consistent chromosomal change in acute promyelocytic leukaemia. Lancet.

[11] Hillestad L. (1957). Acute promyelocytic leukemia. Acta Med. Scand..

[12] Mark M., Chambon P. (2003). Functions of RARs and RXRs in vivo: Genetic dissection of the retinoid signaling pathway. Pure Appl. Chem..

[13] Iwasaki H., Somoza C., Shigematsu H., Duprez E., Iwasaki-Arai J., Mizuno S., Arinobu Y., Geary K., Zhang P., Dayaram T. (2005). Distinctive and indispensable roles of PU.1 in maintenance of hematopoietic stem cells and their differentiation. Blood.

[14] Duprez E., Wagner K., Koch H., Tenen D. (2003). C/EBPbeta: A major PML-RARA-responsive gene in retinoic acid-induced differentiation of APL cells. EMBO J..

[15] Morosetti R., Park D., Chumakov A., Grillier I., Shiohara M., Gombart A., Nakamaki T., Weinberg K., Koeffler H. (1997). A novel, myeloid transcription factor, C/EBPepsilon, is upregulated during granulocytic, but not monocytic, differentiation. Blood.

[16] de Thé H., Lavau C., Marchio A., Chomienne C., Degos L., Dejean A. (1991). The PML-RAR alpha fusion mRNA generated by the t(15;17) translocation in acute promyelocytic leukemia encodes a functionally altered RAR. Cell.

[17] De Braekeleer E., Douet-Guilbert N., De Braekeleer M. (2014). RARA fusion genes in acute promyelocytic leukemia: A review. Expert Rev. Hematol..

[18] Gianni M., Ponzanelli I., Mologni L., Reichert U., Rambaldi A., Terao M., Garattini E. (2000). Retinoid-dependent growth inhibition, differentiation and apoptosis in acute promyelocytic leukemia cells. Expression and activation of caspases. Cell Death Differ..

[19] Breitman T., Selonick S., Collins S. (1980). Induction of differentiation of the human promyelocytic leukemia cell line (HL-60) by retinoic acid. Proc. Natl. Acad. Sci. USA.

[20] Huang M., Ye Y., Chen S., Chai J., Lu J., Zhoa L., Gu L., Wang Z. (1988). Use of all-trans retinoic acid in the treatment of acute promyelocytic leukemia. Blood.

[21] Shen Y., Shen Z., Yan H., Chen J., Zeng X., Li J., Li X., Wu W., Xiong S., Zhao W. (2001). Studies on the clinical efficacy and pharmacokinetics of low-dose arsenic trioxide in the treatment of relapsed acute promyelocytic leukemia: A comparison with conventional dosage. Leukemia.

[22] Shen Z., Shi Z., Fang J., Gu B., Li J., Zhu Y., Shi J., Zheng P., Yan H., Liu Y. (2004). All-trans retinoic acid/As_2_O_3_ combination yields a high quality remission and survival in newly diagnosed acute promyelocytic leukemia. Proc. Natl. Acad. Sci. USA.

[23] de Thé H., Le Bras M., Lallemand-Breitenbach V. (2012). The cell biology of disease: Acute promyelocytic leukemia, arsenic, and PML bodies. J. Cell Biol..

[24] McCulloch D., Brown C., Iland H. (2017). Retinoic acid and arsenic trioxide in the treatment of acute promyelocytic leukemia: Current perspectives. Onco Targets Ther..

[25] Wu Q., Bhole A., Qin H., Karp J., Malek S., Cowell J., Ren M. (2016). SCLLTargeting FGFR1 to suppress leukemogenesis in syndromic and de novo AML in murine models. Oncotarget.

[26] Schlenk R., Dohner K., Kneba M., Götze K., Hartmann F., del Valle F., Kirchen H., Koller E., Fischer J.T., Bullinger L. (2009). Gene mutations and response to treatment with all-trans retinoic acid in elderly patients with acute myeloid leukemia. Results from the AMLSG Trial AML HD98B. Haematologica.

[27] Burnett A.K., Hills R.K., Green C., Jenkinson S., Koo K., Patel Y., Guy C., Gilkes A., Milligan D.W., Goldstone A.H. (2010). The impact on outcome of the addition of all-trans retinoic acid to intensive chemotherapy in younger patients with nonacute promyelocytic acute myeloid leukemia: Overall results and results in genotypic subgroups defined by mutations in NPM1, FLT3, and CEBPA. Blood.

[28] Schlenk R.F., Lübbert M., Benner A., Lamparter A., Krauter J., Herr W., Martin H., Salih H.R., Kündgen A., Horst H.A. (2016). All-trans retinoic acid as adjunct to intensive treatment in younger adult patients with acute myeloid leukemia: Results of the randomized AMLSG 07-04 study. Ann. Hematol..

[29] Watts J., Perez A., Pereira L., Fan Y.-S., Brown G., Vega F., Petrie K., Swords R., Zelent A. (2017). A case of AML characterized by a novel t(4;15)(q31;q22) translocation that confers a growth-stimulatory response to retinoid-based therapy. Int. J. Mol. Sci..

[30] Abe E., Miamura C., Sakagami H., Takeda M., Konno K., Yamazaki T., Yoshiki S., Suda T. (1981). Differentiation of mouse myeloid leukemia cells induced by 1-alpha,25-dihydroxyvitamin D_3_. Proc. Natl. Acad. Sci. USA.

[31] Studzinski G., Bhandal A., Brelvi Z. (1985). Cell cycle sensitivity of HL-60 cells to the differentiation-inducing effects of 1-alpha,25-dihydroxyvitamin D_3_. Cancer Res..

[32] Studzinski G.P., Bhandal A.K., Brelvi Z.S. (1985). A system for monocytic differentiation of leukemic cells HL60 by a short exposure to 1,25-dihydroxycholecalciferol. Proc. Soc. Exp. Biol. Med..

[33] Sharabani H., Izumchenko E., Wang Q., Kreinin R., Steiner M., Barvish Z., Kafka M., Sharoni Y., Levy J., Uskokovic M. (2006). Cooperative antitumor effects of vitamin D_3_ derivatives and rosemary preparations in a mouse model of myeloid leukemia. Int. J. Cancer.

[34] Nachliely M., Sharony E., Bolla N., Kutner A., Danilenko M. (2016). Prodifferentiation Activity of Novel Vitamin D_2_ Analogs PRI-1916 and PRI-1917 and Their Combinations with a Plant Polyphenol in Acute Myeloid Leukemia Cells. Int. J. Mol. Sci..

[35] Chandler P., Buring J., Manson J., Giovannucci E., Moorthy M., Zhang S., Lee I., Lin J. (2015). Circulating Vitamin D Levels and Risk of Colorectal Cancer in Women. Cancer Prev. Res..

[36] Mohr S., Gorham E., Kim J., Hofflich H., Cuomo R., Garland C. (2015). Could vitamin D sufficiency improve the survival of colorectal cancer patients?. J. Steroid Biochem. Mol. Biol..

[37] Mohr S., Gorham E., Kim J., Hofflich H., Garland C. (2014). Meta-analysis of vitamin D sufficiency for improving survival of patients with breast cancer. Anticancer Res..

[38] Maalmi H., Ordóñez-Mena J., Schöttker B., Brenner H. (2014). Serum 25-hydroxyvitamin D levels and survival in colorectal and breast cancer patients: Systematic review and meta-analysis of prospective cohort studies. Eur. J. Cancer.

[39] Muñoz A., Grant W.B. (2022). Vitamin D and Cancer: An Historical Overview of the Epidemiology and Mechanisms. Nutrients.

[40] Carlberg C. (2014). The physiology of vitamin D-far more than calcium and bone. Front. Physiol..

[41] Prosser D., Jones G. (2004). Enzymes involved in the activation and inactivation of vitamin D. Trends Biochem. Sci..

[42] Vaisanen S., Dunlop T., Sinkkonen L., Frank C., Carlberg C. (2005). Spatio-temporal activation of chromatin on the human CYP24 gene promoter in the presence of 1alpha,25-dihydroxyvitamin D_3_. J. Mol. Biol..

[43] Holick M. (1996). Vitamin D and bone health. J. Nutr..

[44] Aranda A., Pascual A. (2001). Nuclear hormone receptors and gene expression. Physiol. Rev..

[45] Pike J., Meyer M. (2014). Fundamentals of vitamin D hormone-regulated gene expression. J. Steroid Biochem. Mol. Biol..

[46] Carlberg C., Seuter S., de Mello V., Schwab U., Voutilainen S., Pulkki K., Nurmi T., Virtanen J., Tuomainen T., Uusitupa M. (2013). Primary vitamin D target genes allow a categorization of possible benefits of vitamin D_3_ supplementation. PLoS ONE.

[47] Gocek E., Kielbinski M., Marcinkowska E. (2007). Activation of intracellular signaling pathways is necessary for an increase in VDR expression and its nuclear translocation. FEBS Lett..

[48] Hsieh J., Dang H., Galligan M., Whitfield G., Haussler C., Jurutka P., Haussler M. (2004). Phosphorylation of human vitamin D receptor serine-182 by PKA suppresses 1,25(OH)_2_D_3_-dependent transactivation. Biochem. Biophys. Res. Commun..

[49] Hsieh J., Jurutka P., Galligan M., Terpening C., Haussler C., Samuels D., Shimizu Y., Shimizu N., Haussler M. (1991). Human vitamin D receptor is selectively phosphorylated by protein kinase C on serine 51, a residue crucial to its trans-activation function. Proc. Natl. Acad. Sci. USA.

[50] Irino S., Taoka T. (1988). Treatment of myelodysplastic syndrome and acute myelogenous leukemia with vitamin D_3_ [1 alpha(OH)D_3_]. Gan Kagaku Ryoho Cancer Chemother..

[51] Nakayama S., Ishikawa T., Yabe H., Nagai K., Kasakura S., Uchino H. (1988). Successful treatment of a patient with acute myeloid leukemia with 1 alpha(OH)D_3_. Nihon Ketsueki Gakkai Zasshi.

[52] Hellström E., Robèrt K., Gahrton G., Mellstedt H., Lindemalm C., Einhorn S., Björkholm M., Grimfors G., Udén A., Samuelsson J. (1988). Therapeutic effects of low-dose cytosine arabinoside, alpha-interferon, 1 alpha-hydroxyvitamin D_3_ and retinoic acid in acute leukemia and myelodysplastic syndromes. Eur. J. Haematol..

[53] Hellström E., Robèrt K., Samuelsson J., Lindemalm C., Grimfors G., Kimby E., Oberg G., Winqvist I., Billström R., Carneskog J. (1990). Treatment of myelodysplastic syndromes with retinoic acid and 1 alpha-hydroxy-vitamin D_3_ in combination with low-dose ara-C is not superior to ara-C alone. Results from a randomized study. The Scandinavian Myelodysplasia Group (SMG). Eur. J. Haematol..

[54] Ferrero D., Campa E., Dellacasa C., Campana S., Foli C., Boccadoro M. (2004). Differentiating agents + low-dose chemotherapy in the management of old/poor prognosis patients with acute myeloid leukemia or myelodysplastic syndrome. Haematologica.

[55] Donovan P.J., Sundac L., Pretorius C.J., d’Emden M.C., McLeod D.S.A. (2013). Calcitriol-Mediated Hypercalcemia: Causes and Course in 101 Patients. J. Clin. Endocrinol. Metab..

[56] Motomura S., Kanamori H., Maruta A., Kodama F., Ohkubo T. (1991). The effect of 1-hydroxyvitamin D_3_ for prolongation of leukemic transformation-free survival in myelodysplastic syndromes. Am. J. Hematol..

[57] Mellibovsky L., Díez A., Pérez-Vila E., Serrano S., Nacher M., Aubía J., Supervía A., Recker R. (1998). Vitamin D treatment in myelodysplastic syndromes. Br. J. Haematol..

[58] Hathcock J.N., Shao A., Vieth R., Heaney R. (2007). Risk assessment for vitamin D. Am. J. Clin. Nutr..

[59] Nadkarni S., Chodynski M., Corcoran A., Marcinkowska E., Brown G., Kutner A. (2015). Double point modified analogs of vitamin D as potent activators of vitamin D receptor. Curr. Pharm. Des..

[60] Norman A.W., Zhou J.Y., Henry H.L., Uskokovic M.R., Koeffler H.P. (1990). Structure-function studies on analogues of 1 alpha,25-dihydroxyvitamin D_3_: Differential effects on leukemic cell growth, differentiation, and intestinal calcium absorption. Cancer Res..

[61] Zmijewski M.A., Carlberg C. (2020). Vitamin D receptor(s): In the nucleus but also at membranes?. Exp. Dermatol..

[62] Maestro M.A., Molnár F., Carlberg C. (2019). Vitamin D and Its Synthetic Analogs. J. Med. Chem..

[63] Gaikwad S., González C.M., Vilariño D., Lasanta G., Villaverde C., Mouriño A., Verlinden L., Verstuyf A., Peluso-Iltis C., Rochel N. (2021). Lithocholic acid-based design of noncalcemic vitamin D receptor agonists. Bioorg. Chem..

[64] González C.M., Gaikwad S., Lasanta G., Loureiro J., Nilsson N., Peluso-Iltis C., Rochel N., Mouriño A. (2021). Design, synthesis and evaluation of side-chain hydroxylated derivatives of lithocholic acid as potent agonists of the vitamin D receptor (VDR). Bioorg. Chem..

[65] Sasaki H., Masuno H., Kawasaki H., Yoshihara A., Numoto N., Ito N., Ishida H., Yamamoto K., Hirata N., Kanda Y. (2020). Lithocholic Acid Derivatives as Potent Vitamin D Receptor Agonists. J. Med. Chem..

[66] Harrison J., Bershadskiy A. (2012). Clinical experience using vitamin D and analogs in the treatment of myelodysplasia and acute myeloid leukemia: A review of the literature. Leuk. Res. Treat..

[67] Bennett J., Catovsky D., Daniel M., Flandrin G., Galton D., Gralnick H., Sultan C. (1976). Proposals for the classification of the acute leukaemias. French-American-British (FAB) co-operative group. Br. J. Haematol..

[68] Vardiman J.W., Thiele J., Arber D.A., Brunning R.D., Borowitz M.J., Porwit A., Harris N.L., Le Beau M.M., Hellström-Lindberg E., Tefferi A. (2009). The 2008 revision of the World Health Organization (WHO) classification of myeloid neoplasms and acute leukemia: Rationale and important changes. Blood.

[69] Gralnick H.R., Galton D.A.G., Catovsky D., Sultan C., Bennett J.M. (1977). Classification of Acute Leukemia. Ann. Intern. Med..

[70] Vardiman J., Harris N., Brunning R. (2002). The World Health Organization (WHO) classification of the myeloid neoplasms. Blood.

[71] Li S., Mason C.E., Melnick A. (2016). Genetic and epigenetic heterogeneity in acute myeloid leukemia. Curr. Opin. Genet. Dev..

[72] Baurska H., Kiełbiński M., Biecek P., Haus O., Jaźwiec B., Kutner A., Marcinkowska E. (2014). Monocytic differentiation induced by side-chain modified analogs of vitamin D in ex vivo cells from patients with acute myeloid leukemia. Leuk. Res..

[73] Gocek E., Kielbinski M., Baurska H., Haus O., Kutner A., Marcinkowska E. (2010). Different susceptibilities to 1,25-dihydroxyvitamin D_3_-induced differentiation of AML cells carrying various mutations. Leuk. Res..

[74] Gocek E., Marchwicka A., Baurska H., Chrobak A., Marcinkowska E. (2012). Opposite regulation of vitamin D receptor by ATRA in AML cells susceptible and resistant to vitamin D-induced differentiation. J. Steroid Biochem. Mol. Biol..

[75] Paubelle E., Zylbersztejn F., Maciel T., Carvalho C., Mupo A., Cheok M., Lieben L., Sujobert P., Decroocq J., Yokoyama A. (2020). Vitamin D Receptor Controls Cell Stemness in Acute Myeloid Leukemia and in Normal Bone Marrow. Cell Rep..

[76] Munker R., Norman A., Koeffler H. (1986). Vitamin D compounds. Effect on clonal proliferation and differentiation of human myeloid cells. J. Clin. Investig..

[77] Sohal J., Chase A., Mould S., Corcoran M., Oscier D., Iqbal S., Parker S., Welborn J., Harris R., Martinelli G. (2001). Identification of four new translocations involving FGFR1 in myeloid disorders. Genes Chromosomes Cancer.

[78] Gu T., Goss V., Reeves C., Popova L., Nardone J., Macneill J., Walters D., Wang Y., Rush J., Comb M. (2006). Phosphotyrosine profiling identifies the KG-1 cell line as a model for the study of FGFR1 fusions in acute myeloid leukemia. Blood.

[79] Jin Y., Zhen Y., Haugsten E., Wiedlocha A. (2011). The driver of malignancy in KG-1a leukemic cells, FGFR1OP2–FGFR1, encodes an HSP90 addicted oncoprotein. Cell. Signal..

[80] Marchwicka A., Corcoran A., Berkowska K., Marcinkowska E. (2016). Restored expression of vitamin D receptor and sensitivity to 1,25-dihydroxyvitamin D_3_ in response to disrupted fusion FOP2-FGFR1 gene in acute myeloid leukemia cells. Cell Biosci..

[81] Jackson C., Medeiros L., Miranda R. (2010). 8p11 myeloproliferative syndrome: A review. Hum. Pathol..

[82] Liu J.J., Meng L. (2018). 8p11 Myeloproliferative syndrome with t(8;22)(p11;q11): A case report. Exp. Ther. Med..

[83] Boutzen H., Saland E., Larrue C., de Toni F., Gales L., Castelli F.A., Cathebas M., Zaghdoudi S., Stuani L., Kaoma T. (2016). Isocitrate dehydrogenase 1 mutations prime the all-trans retinoic acid myeloid differentiation pathway in acute myeloid leukemia. J. Exp. Med..

[84] Sabatier M., Boet E., Zaghdoudi S., Guiraud N., Hucteau A., Polley N., Cognet G., Saland E., Lauture L., Farge T. (2021). Activation of Vitamin D Receptor Pathway Enhances Differentiating Capacity in Acute Myeloid Leukemia with Isocitrate Dehydrogenase Mutations. Cancers.

[85] Manfredini R., Trevisan F., Grande A., Tagliafico E., Montanari M., Lemoli R., Visani G., Tura S., Ferrari S., Ferrari S. (1999). Induction of a functional vitamin D receptor in all-trans-retinoic acid-induced monocytic differentiation of M2-type leukemic blast cells. Cancer Res..

[86] Marchwicka A., Cebrat M., Łaszkiewicz A., Śnieżewski Ł., Brown G., Marcinkowska E. (2016). Regulation of vitamin D receptor expression by retinoic acid receptor alpha in acute myeloid leukemia cells. J. Steroid Biochem. Mol. Biol..

[87] Katoh M., Nakagama H. (2014). FGF receptors: Cancer biology and therapeutics. Med. Res. Rev..

[88] Katoh M. (2016). FGFR inhibitors: Effects on cancer cells, tumor microenvironment and whole-body homeostasis (Review). Int. J. Mol. Med..

[89] Marchwicka A., Jakuszak A., Grembowska A., Kumari P., Marcinkowska E. (2021). The Influence of Overexpressed Fibroblast Growth Factor Receptors Towards Vitamin D Receptor Expression and Activity. Preprints.

[90] Xie Y., Su N., Yang J., Tan Q., Huang S., Jin M., Ni Z., Zhang B., Zhang D., Luo F. (2020). FGF/FGFR signaling in health and disease. Signal. Transduct. Target. Ther..

[91] Eswarakumar V.P., Lax I., Schlessinger J. (2005). Cellular signaling by fibroblast growth factor receptors. Cytokine Growth Factor Rev..

[92] Wang X., Rao J., Studzinski G.P. (2000). Inhibition of p38 MAP kinase activity up-regulates multiple MAP kinase pathways and potentiates 1,25-dihydroxyvitamin D_3_-induced differentiation of human leukemia HL60 cells. Exp. Cell Res..

[93] Wang X., Studzinski G. (2001). Inhibition of p38 MAP kinase potentiates the JNK/SAPK pathway and AP-1 activity in monocytic but not in macrophage or granulocytic differentiation of HL60 cells. J. Cell Biochem..

[94] Hart K., Robertson S., Kanemitsu M., Meyer A., Tynan J., Donoghue D. (2000). Transformation and Stat activation by derivatives of FGFR1, FGFR3, and FGFR4. Oncogene.

[95] Sasieni P.D., Shelton J., Ormiston-Smith N., Thomson C.S., Silcocks P.B. (2011). What is the lifetime risk of developing cancer? The effect of adjusting for multiple primaries. Br. J. Cancer.

